# Supplementary material for: Mechanisms of gene regulation by SRCAP and H2A.Z
Source: Nat Commun. 2026 Mar 6;17:3560. doi: 10.1038/s41467-026-70087-x (PMC13087030; doi:10.1038/s41467-026-70087-x)
Supplement: Supplementary file 1 — Supplementary Information [file 41467_2026_70087_MOESM1_ESM.pdf]

## Supplementary Figures

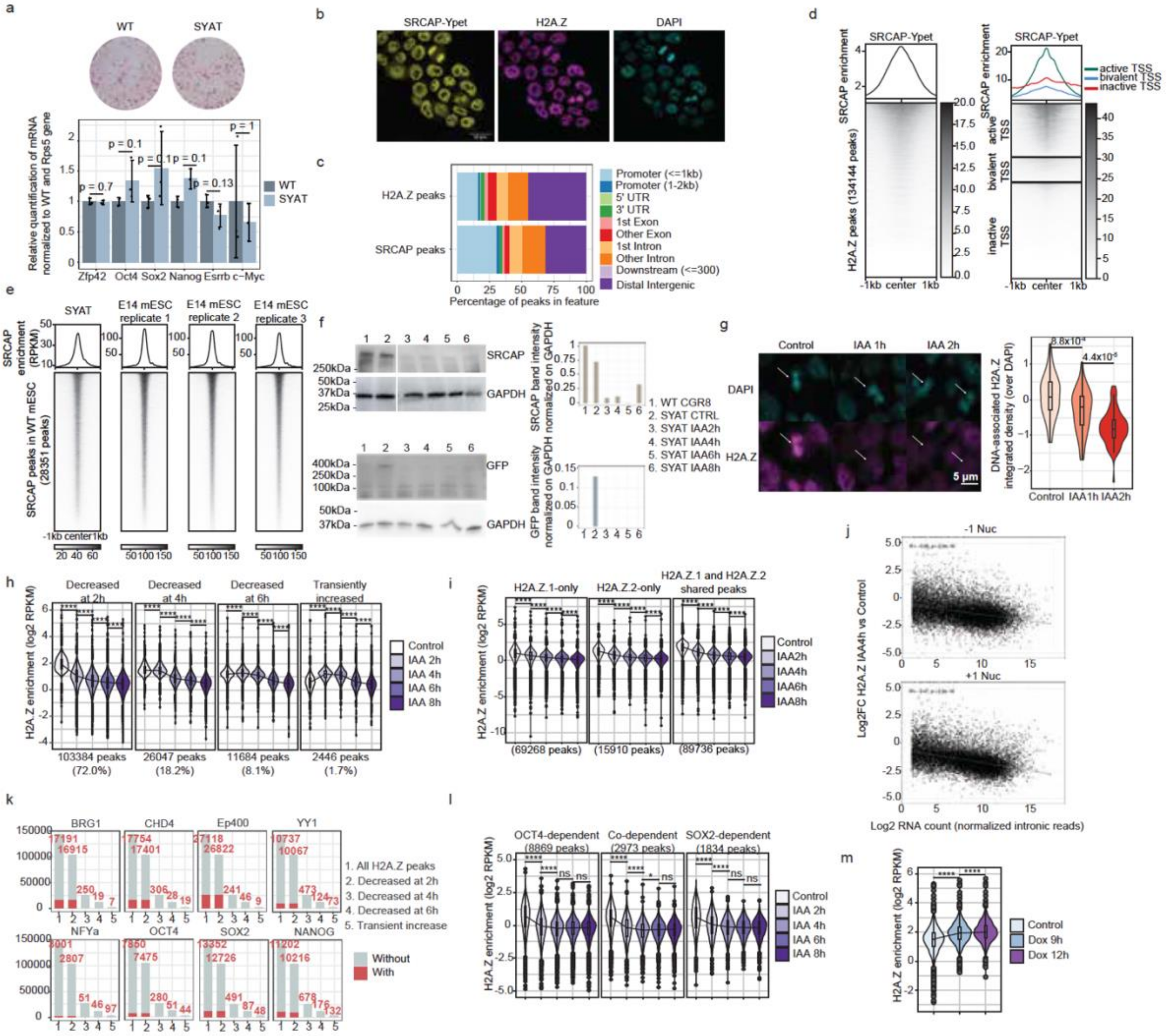


**Figure S1**: Related to Fig.1. **a**, Validation of pluripotency of SYAT cells. Top: Alkaline Phosphatase staining of wt and SYAT mESCs. Bottom: Quantification of pluripotency TF expression by RT-qPCR in wt and SYAT mESCs. N=3. p-values were determined by a two-sided Kruskal-Wallis test. **b**, Microscopy snapshot of SYAT cells stained with an anti-H2A.Z antibody and DAPI. **c**, SRCAP and H2A.Z peak enrichment by genomic locus type. **d**, SRCAP enrichment determined by CUT&Tag with an anti-GFP antibody at H2A.Z peaks (left) or TSS (right). e, SRCAP enrichment in SYAT and wt E14 mESCs at SRCAP peaks detected in wt mESCs (data from [32]). **f**, Western Blot on whole cellular extract from wt CGR8 mESCs or SYAT treated or not with IAA. Up : Membrane was stained with SRCAP and GAPDH antibodies. Down : Membrane was stained with an anti-GFP and anti-GAPDH antibodies. Respective GAPDH-normalized signals were quantified on the left. **g**, Left: Microscopy snapshots of SYAT cells treated with control or IAA for 2 h and stained with anti-H2A.Z antibody and DAPI. White arrows: mitotic cells. Right: Quantification of DNA-associated H2A.Z normalized on DAPI in mitotic cells treated with IAA or control. N = 50 cells per condition. p-values, determined by a two-sided pairwise Wilcoxon test, are indicated on the plot. Boxes: interquartile range (IQR); whiskers: 1.5xIQR; dots: outlier points. **h**, Classification of H2A.Z-enriched regions as a function of H2A.Z turnover rate. Black line: changes in median values. Boxes: interquartile range (IQR); whiskers: 1.5xIQR; dots: outlier points. Exact p-values are provided in supplementary Table 7. **i**, H2A.Z enrichment in SYAT cells treated with control or IAA for 2, 4, 6, or 8 h, at genomic regions enriched for H2A.Z.1, H2A.Z.2 or both. Black line: changes in median values. Boxes: interquartile range (IQR); whiskers: 1.5xIQR; dots: outlier points. Exact p-values are provided in supplementary Table 7. **j**, Changes of H2A.Z levels at the -1 and +1 nucleosomes, between 0 and 4 h of IAA treatment, as a function of intronic reads of the downstream genes in the absence of IAA. **k**, Total (gray) or enriched

(red) peaks for BRG1, CHD4 and Ep400 in the different clusters defined in (**d**). **l**, H2A.Z enrichment at regions depending on OCT4 or SOX2 for their accessibility. Black line: changes in median values. Boxes: interquartile range (IQR); whiskers: 1.5xIQR; dots: outlier points. p-values were determined by a two-sided pairwise Wilcoxon test, *: $p<0.05$, ****: $p<0.0001$, ns: non-significant. Exact p-values are provided in supplementary Table 7. **m**, H2A.Z enrichment peaks enriched for H2A.Z and Oct4 (1989 peaks) upon OCT4 knockdown in ZHBTc4 treated with Doxycycline for 9 or 12 h. Black line: changes in median values. Boxes: interquartile range (IQR); whiskers: 1.5xIQR; dots: outlier points. ****: $p<0.0001$, *: $p<0.05$, ns: non-significant. Exact p-values are provided in supplementary Table 7.

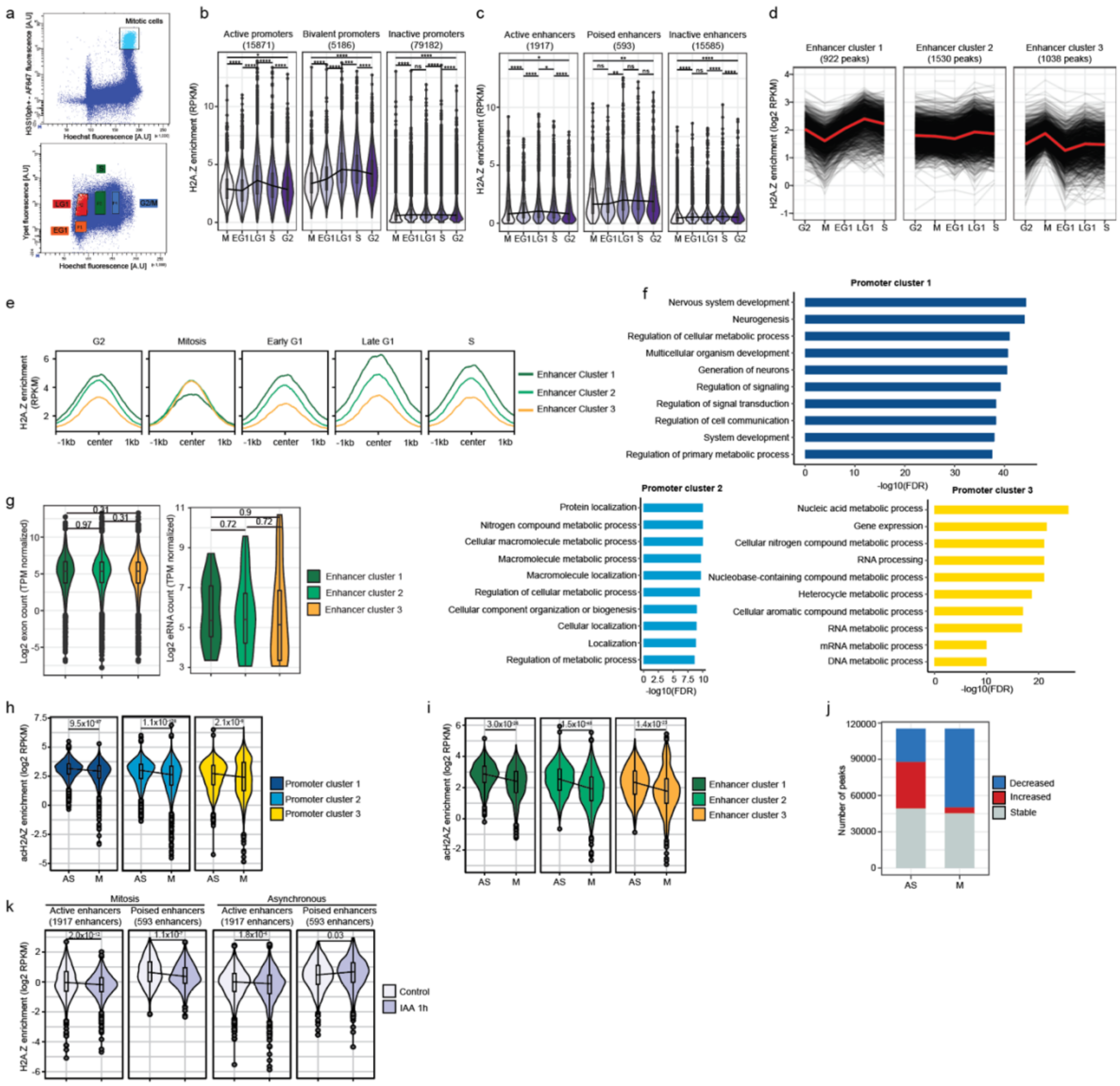

**Figure S2:** Related to Fig.2. **a**, FACS-plot from cell sorting of mitotic cells (upper panel) or EG1, LG1, S and G2 phase cells. **b** and **c**, RPKM-normalized H2A.Z scores across the cell cycle for all promoters (**b**) and enhancers (**c**). Black line: changes in median values. Boxes: interquartile range (IQR); whiskers: 1.5xIQR; dots: outlier points. p-values were determined by a pairwise Wilcoxon test, ****: $p<0.0001$, ***: $p<0.001$, **: $p<0.01$, *: $p<0.05$, ns: non-significant. Exact p-values are provided in Supplementary Table 7. **d**, H2A.Z enrichment in enhancer peaks with decreased, stable or increased mitotic H2A.Z levels with respect to G2 and EG1 phases. Red line: changes in median values. **e**, H2A.Z enrichment around H2A.Z peaks in the clusters defined in (**d**). **f**, Top-ten enriched GO terms in mitotic promoter clusters. **g**, Intronic counts of genes regulated by each of the three enhancer clusters (left) and enhancer RNA counts (right) of the enhancers belonging to the three mitotic clusters. Data from two independent biological RNA-Seq replicates were used. Boxes: interquartile range (IQR); whiskers: 1.5xIQR; dots: outlier points. p-values from two-sided pairwise Wilcoxon test are indicated on the plot. **h** and **i**, acH2A.Z enrichment in asynchronous and mitotic cells in mitotic promoter (**h**) and enhancer (**i**) clusters. Black line: changes in median values. Boxes: interquartile range (IQR); whiskers: 1.5xIQR; dots: outlier points. p-values from two-sided pairwise Wilcoxon test are indicated on the plot. **j**, Numbers of peaks displaying increased, stable or decreased H2A.Z score after 1 h of IAA treatment in asynchronous or mitotic cells. **k**, H2A.Z enrichment at enhancers in mitotic or asynchronous SYAT cells treated for 1 h with IAA. Black line: changes in median values. Boxes: interquartile range (IQR); whiskers: 1.5xIQR; dots: outlier points. p-values from two-sided pairwise Wilcoxon test are indicated on the plot.

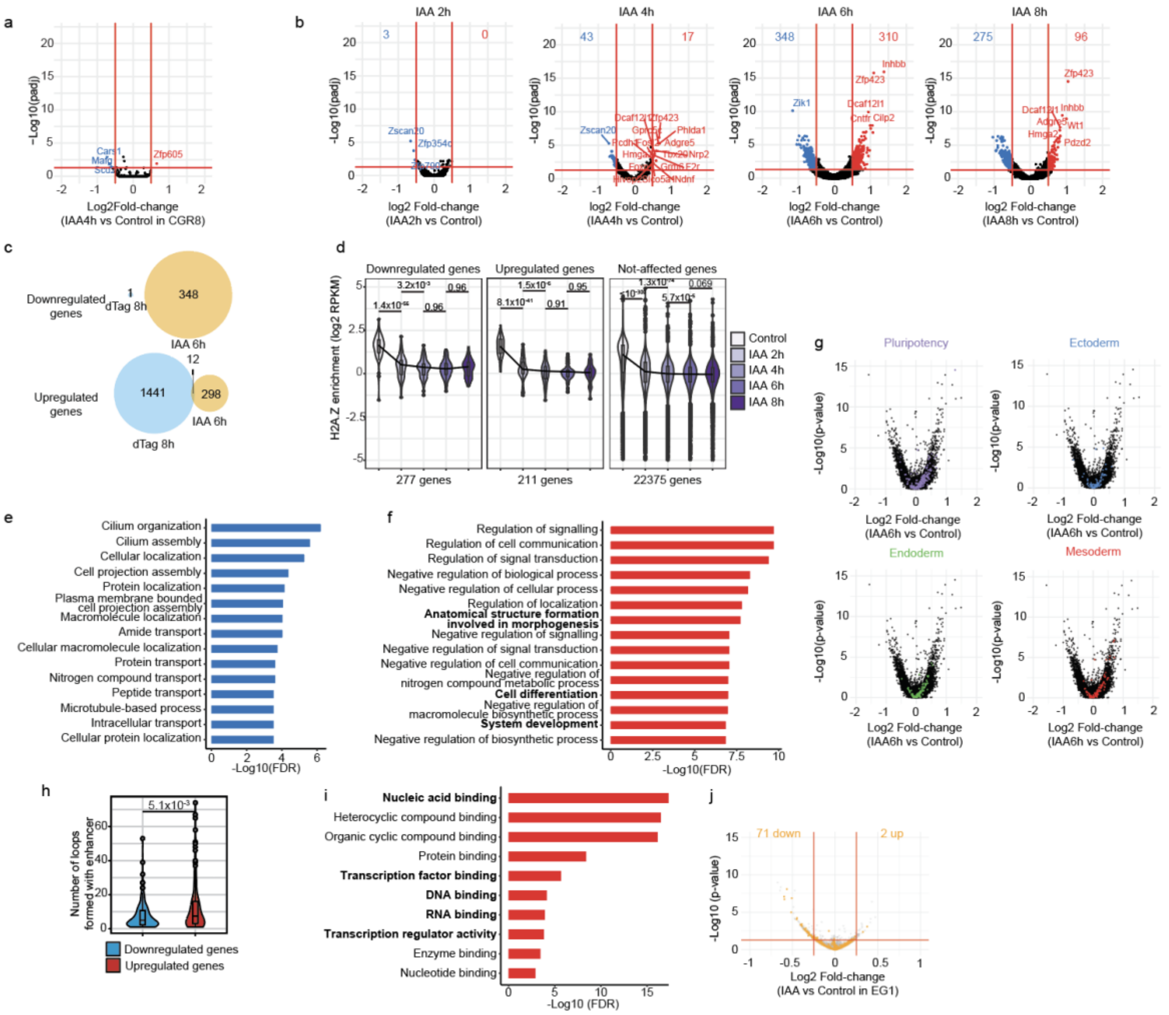

**Figure S3:** Related to Fig.3. **a**, Fold-change of intronic reads and adjusted p-value, for wt cells treated for 4 h with IAA Data from two independent biological RNA-Seq replicates were used. **b**, Fold-change of exonic reads and adjusted p-value, for SYAT cells treated 2, 4, 6 and 8 h with IAA. Numbers: genes significantly downregulated (blue) or upregulated (red) with a threshold of |log2FC|>0.5 and adjusted p-value < 0.05. Data from two independent biological RNA-Seq replicates were used. **c**, Genes overlapping between those significantly affected after 6 h of IAA treatment and genes affected after 8 h of H2A.Z.1 depletion in [20] when analyzing exonic reads. **d**, H2A.Z enrichment (Log2 RPKM) determined by ChIP-Seq, at promoters of upregulated or downregulated genes after 6 h of IAA treatment, or unaffected genes over the course of 8 h of IAA treatment. p-values were determined by a two-sided pairwise Wilcoxon test. Black line: changes in median values. Boxes: interquartile range (IQR); whiskers: 1.5xIQR; dots: outlier points. **e** and **f**, Top 15 GO terms enriched for downregulated (**e**) or upregulated (**f**) genes after 6 h of IAA treatment in the "cellular process" category. **g**, Fold-change of intronic reads and adjusted p-value for SYAT cells treated for 6 h with IAA. Genes associated with pluripotency or commitment to the three germ layers are colored. **h**, Numbers of loops formed between one given promoter of upregulated or downregulated genes and enhancers (data from Enhancer Atlas). Boxes: interquartile range (IQR); whiskers: 1.5xIQR; dots: outlier points. p-values were determined by a two-sided pairwise Wilcoxon test. **i**, Top-ten enriched GO terms for molecular functions, in proteins increased in the proteome upon 4 h of IAA treatment. **j**, Fold-change of intronic reads for SYAT cells treated for 1 h with IAA and sorted in early G1. Housekeeping genes are colored in orange. Numbers: housekeeping genes significantly downregulated or upregulated with a threshold of |log2FC|>0.25 and p-value < 0.05.

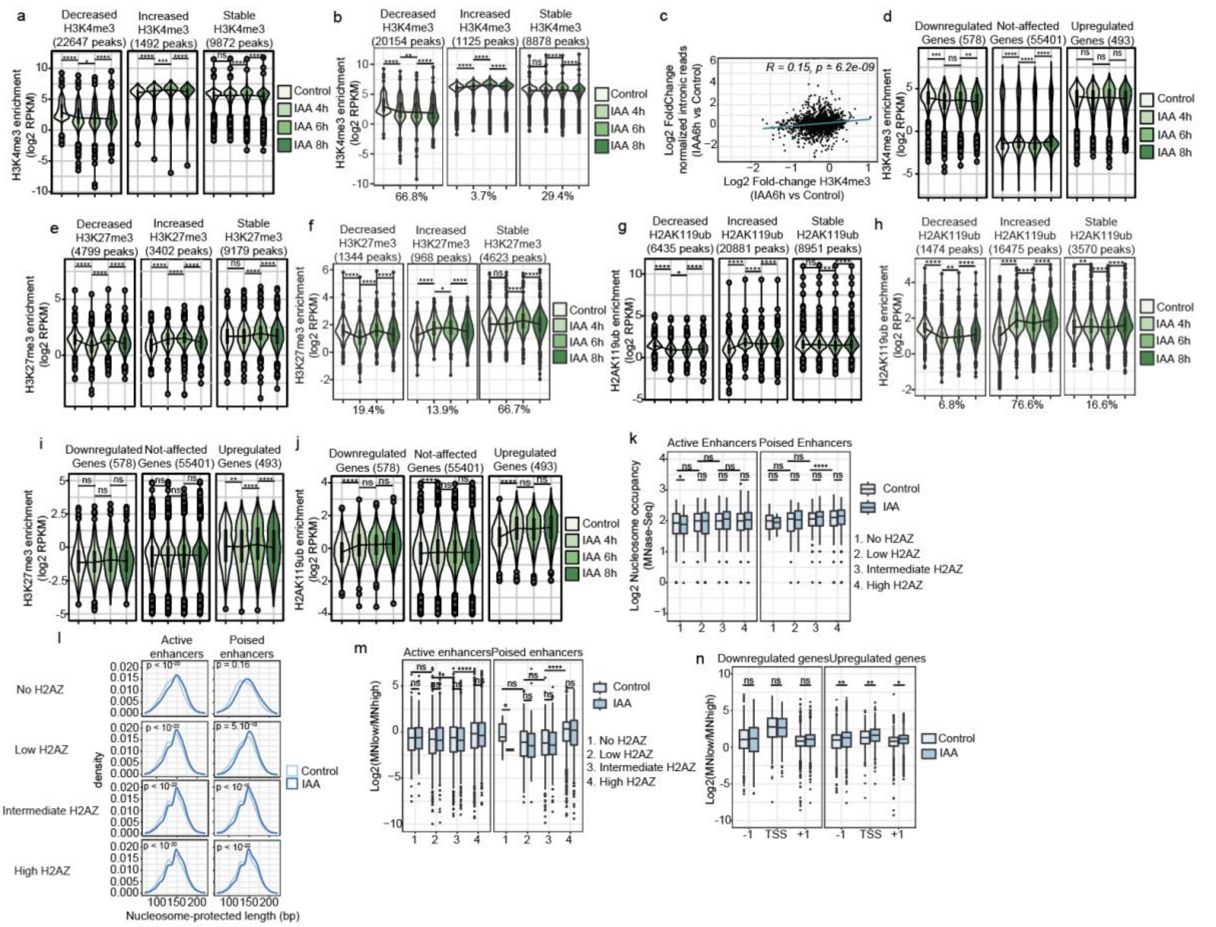

**Figure S4**: Related to Fig.4. **a**-b, Enrichment of H3K4me3 in SYAT cells treated with control or IAA for 4, 6 and 8 h in all H3K4me3 peaks (a) or H3K4me3 peaks also enriched for H2A.Z (b). **c**, Changes in intronic reads of genes controlled by a bivalent promoter as a function of H3K4me3 enrichment between 6 h of IAA treatment and control. Upper right corner: Pearson correlation coefficient. **d**, H3K4me3 enrichment in SYAT cells treated with control or IAA for 4, 6 and 8 h, at promoters of genes downregulated or upregulated upon SRCAP depletion. **e-h**, Enrichment of H3K27me3 at all H3K27me3 peaks (e) or peaks shared with H2A.Z (f) and H2AK119ub1 at all H2AK119ub1 peaks (g) or peaks shared with H2A.Z (h) in SYAT cells treated with control or IAA for 4, 6 and 8 h. **i** and **j**, H3K27me3 (**i**) and H2AK119ub1 (**j**) enrichment in SYAT cells treated with control or IAA for 4, 6 and 8 h, at promoters of genes downregulated or upregulated upon SRCAP depletion. **k**, Nucleosome occupancy at enhancers in SYAT cells as a function of H2A.Z levels, in control or after 4 h of IAA treatment. The numbers of analyzed nucleosome are provided in supplementary Table 6. **l**, MNase-Seq fragment densities in SYAT cells treated or not with IAA for 4 h as a function of H2A.Z enrichment and enhancer type. **m**, Fragile nucleosomes located at different types of enhancers, as a function of H2A.Z levels. The numbers of analyzed nucleosome are provided in supplementary Table 6 **n**, Fragile nucleosomes at -1, TSS and +1 nucleosomes of genes downregulated and upregulated upon SRCAP degradation. The numbers of analyzed nucleosome are provided in supplementary Table 6 p-values comparing H2A.Z levels in boxplot were determined by a two-sided pairwise Wilcoxon test. p-values comparing CTRL and IAA conditions in boxplot were determined by a two-sided Welch two samples t-test. p-values comparing fragment length distributions were determined by a Kolmogorov-Smirnov test. ****: $p<0.0001$; ***: $p<0.001$; **: $p<0.01$; *: $p<0.05$, ns: non-significant. Exact p-values are provided in supplementary table 7. For all violin-plots and boxplots, Black line: changes in median values. Boxes: interquartile range (IQR); whiskers: 1.5xIQR; dots: outlier points.

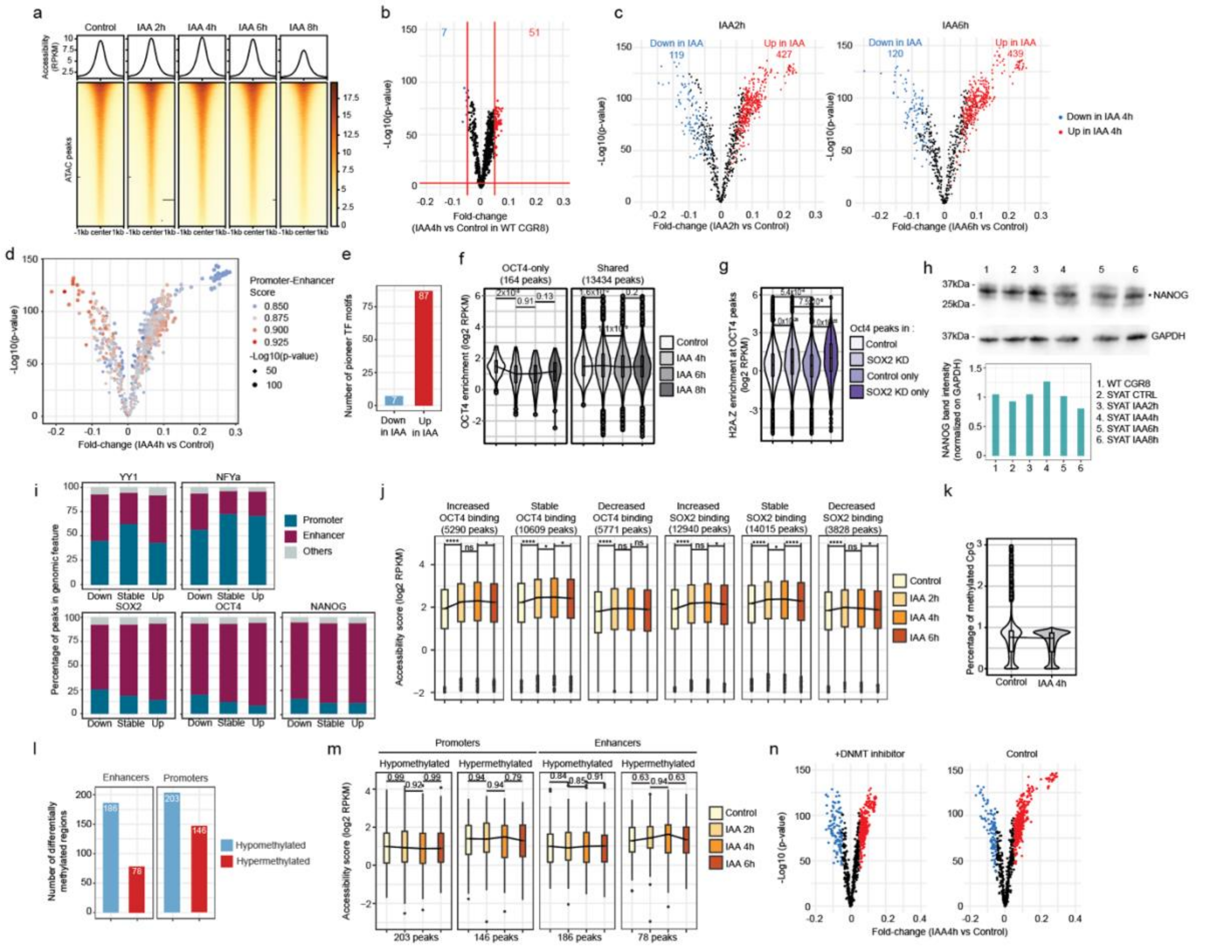


**Figure S5:** Related to Fig.5. **a**, Heatmap of chromatin accessibility determined by ATAC-Seq and centered on the summit of accessible peaks, in SYAT cells upon IAA treatment for 2, 4, 6 and 8 h. **b**, Fold-change of TF footprinting calculated using TOBIAS in wt CGR8 cells treated with control or IAA. Colors represent TF motifs predicted to lose (in blue) or gain (in red) binding upon IAA treatment in CGR8 cells with a threshold of |FC|>0.05 and p-value < 0.0001. **c**, Fold-change of TF footprinting calculated using TOBIAS in SYAT cells treated 2 h (left panel) or 6 h (right panel) with IAA versus control. Colors: TF motifs predicted to lose (blue) or gain (red) binding upon 4 h-IAA treatment with a threshold of |FC|>0.05 and p-value < 0.0001. **d**, Fold-change of TF footprinting calculated using TOBIAS in SYAT cells treated 4 h with IAA versus control. Color scale: promoter-enhancer score; dot size: -log10(p-value). **e**, Number of binding motifs of pTFs, among motifs predicted to lose or gain binding upon 4 h of IAA treatment. **f**, OCT4 ChIP-seq enrichment score at peaks bound by OCT4 only or shared with SOX2 in SYAT cells treated with IAA for 4, 6 and 8 h or control. p-values represented on the plot were determined by a two-sided pairwise Wilcoxon test. **g**, H2A.Z enrichment score in 2TS22C cells, at all peaks bound by OCT4 in control conditions, upon SOX2 knockdown, or at peaks unique to control or SOX2 knockdown conditions. p-values represented on the plot were determined by a two-sided pairwise Wilcoxon test. **h**, Western Blot displaying NANOG level in wt mESCs or SYAT cells treated with IAA for 2, 4, 6 or 8 hours. The star in NANOG blot correspond to GAPDH as the membrane was stained with an anti-GAPDH before. Histogram represents quantification of NANOG protein over GAPDH signal. **i**, Percentage of peaks where TFs increase, decrease or maintain a stable binding upon IAA treatment, in promoters or enhancers. **j**, Accessibility changes upon IAA treatment at peaks gaining, losing or maintaining binding of OCT4 or SOX2. p-values represented on the plot were determined by a two-sided pairwise Wilcoxon test; ****: p<0.0001; *: p<0.05, ns: non-significant. **k**, Percentage of methylated cytosines in H2A.Z peaks in SYAT cells treated with control or IAA. **l**, Number of promoters or enhancers with cytosine hyper- or hypomethylation upon 4 h of IAA treatment. **m**, Chromatin accessibility changes upon IAA treatment at hyper- or hypomethylated promoters and enhancers. p-values represented on the plot were determined by a two-sided pairwise Wilcoxon test. **n**, Fold-change of TF footprinting calculated using TOBIAS, in SYAT cells treated or not for 5 days with a DNMT1 inhibitor and treated 4 h with IAA versus control.

For all violin-plots and boxplots, Black line: changes in median values. Boxes: interquartile range (IQR); whiskers: 1.5xIQR; dots: outlier points.

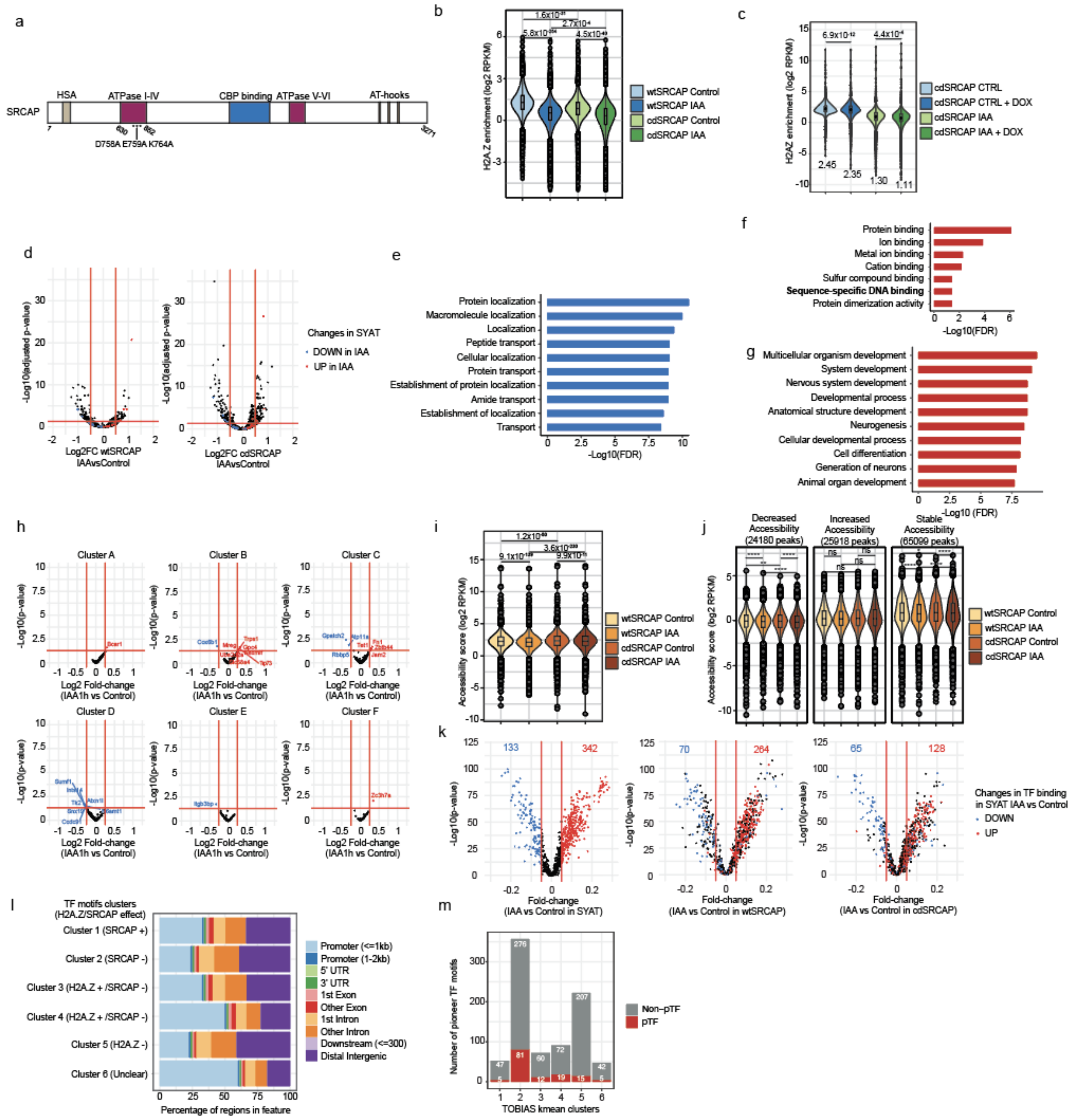

**Figure S6:** Related to Fig.6. **a**, Schematic representation of the SRCAP protein with its functional domains. Stars: mutations introduced in the ATPase domain of SRCAP. **b**, H2A.Z enrichment determined by ChIP-Seq in all H2A.Z peaks (115189 peaks) upon overexpression of wtSRCAP or cdSRCAP and treated with control or IAA for 4 h. p-values were determined by two-sided Welch two samples t-test. **c**, H2A.Z enrichment determined by ChIP-Seq in all H2A.Z peaks (115189 peaks) in cells overexpressing (+DOX) or not cdSRCAP and depleted (IAA) or not (CTRL) for endogenous SRCAP. p-values were determined by two-sided Welch two samples t-test. **d**, Fold-change in intronic reads (2 independent biological replicates) in wtSRCAP (left panel) or cdSRCAP (right panel) treated with control or IAA for 4 h. Colors: intronic read changes observed in SYAT cells after 4 h of IAA treatment. **e**, Top 10 GO terms in the “cellular process” category, enriched for genes in cluster D and E defined in Fig.6c. **f**, Top 10 GO terms in the “molecular function” category, enriched for genes upregulated in cdSRCAP IAA compared to wtSRCAP IAA. **g**, Top 10 GO terms in the “cellular process” category, enriched for genes upregulated in cdSRCAP IAA compared to wtSRCAP IAA. **h**, Fold-change in intronic reads in the 6 clusters defined in Fig.6f for SYAT cells treated for 1 h with IAA or control and FACSorted in LG1 (2 independent biological replicates). **i**, Chromatin accessibility changes in all accessible peaks (65630 peaks) upon overexpression of wtSRCAP or cdSRCAP and treated with control or IAA for 4 h. p-values were determined by two-sided pairwise Wilcoxon rank sum tests.  **j**, Chromatin accessibility changes upon overexpression of wtSRCAP or cdSRCAP and treated

with control or IAA for 4 h, in peaks gaining, losing, or maintaining accessibility in SYAT cells upon 4 h of IAA treatment. p-values were determined by two-sided pairwise Wilcoxon rank sum tests and are provided in supplementary Table 7. ****: p<0.0001; **: p<0.01; *: p<0.05, ns: non-significant. **k**, Fold-change of TF footprinting in regions rescued for H2A.Z levels calculated using TOBIAS in SYAT, wtSRCAP and cdSRCAP cells treated with control or IAA for 4 h. Colored dots: motifs predicted to gain or lose binding in rescued regions in SYAT cells treated with IAA versus control. Numbers: Motifs significantly up or down in SYAT and that are still up or down in wtSRCAP or cdSRCAP. **l**, Percentage of motif occurrence, belonging to the six clusters determined in Fig.6h, in different types of genomic regions. **m**, Number of binding motifs of pioneer TFs in the six motif clusters determined in Fig.6h. For all violin-plots, boxes: interquartile range (IQR); whiskers: 1.5xIQR; dots: outlier points.

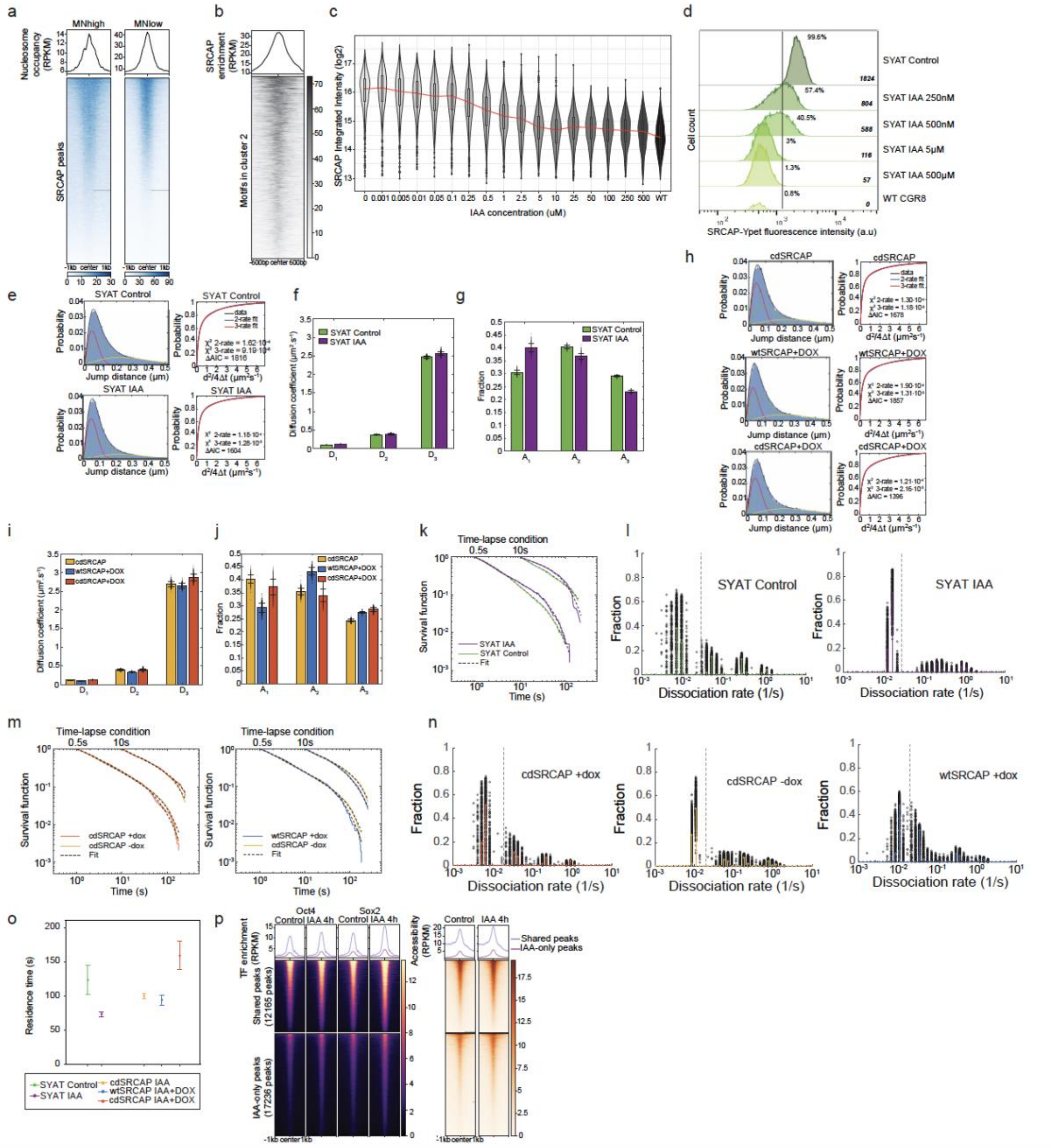

**Figure S7:** Related to Fig.7. **a,** Nucleosome occupancy at SRCAP peaks (36780 peaks), determined by MNase-Seq using either high (left) or low (right) concentration of MNase. **b,** SRCAP enrichment assessed by CUT&RUN in a 1.2kb region centered around TF motifs belonging to cluster 2 (29552 regions) (Fig.6h). **c,** Quantification of SRCAP-Ypet fluorescence intensity after background subtraction by live-imaging in SYAT cells treated for 2h with different doses of IAA or CTRL. **d,** SRCAP-Ypet fluorescence intensity determined by Flow Cytometry analysis of SYAT cells treated for 2h with different doses of IAA or CTRL. Percentage of Ypet positive cells as well as mean intensity after subtraction of Ypet intensity in WT CGR8 cells are indicated. **e,** Jump distance distributions (left) and cumulative jump distance distributions (right) of Halo-NANOG in CTRL or after 4h IAA treatment. Jump distance distributions are overlayed with a three-component diffusion model (black) and the three single components (red, green, yellow). Cumulative jump distance distributions show data (black line) fitted with a 2-component (blue) and a 3-component (red) diffusion model. The insets give values for reduced $\chi^2$ and Akaike Information Criterion (AIC), revealing the better suitability of the 3-component fit. CTRL: 29891 tracks in 25 cells, IAA: 14294 tracks in 22 cells. **f** and **g,** Diffusion coefficients (f) and fractions (g) of three-component diffusion model fitted to (e). Data represent mean values ± s.d. from 400

resamplings with randomly selected 80 % of the data. **h,** Jump distance distributions (left) and cumulative jump distance distributions (right) of Halo-NANOG upon IAA treatment in cdSRCAP cells, wtSRCAP cells treated with Dox and in cdSRCAP cells treated with Dox. Data displayed as in e. wtSRCAP+dox: 6807 tracks in 11 cells, cdSRCAP +dox: 7207 tracks in 19 cells, cdSRCAP-dox: 9244 tracks in 34 cells. **i** and **j,** Diffusion coefficients (i) and fractions (j) of three-component diffusion model fitted to (h). Data represent mean values ± s.d. from 400 resamplings with randomly selected 80 % of the data. **k,** Survival time distributions of Halo-NANOG in SYAT CTRL (green) or treated with IAA (purple) at time-lapse conditions shown on top and survival time functions obtained with GRID (black dashed lines). IAA: 1121 tracks in 26 cells, CTRL: 2813 tracks in 16 cells. **l,** State spectra of dissociation rates obtained with GRID from distributions in (k) using all data for Halo-NANOG in SYAT CTRL (green, left panel) or treated with IAA (purple, right panel). As an error estimation GRID was run 500 times with each run using 80 % of the data (black circles). The dashed lines indicate a dissociation rate of 0.02 $s^{-1}$. **m,** Survival time distributions of Halo-NANOG in cdSRCAP cells +dox (red), in cdSRCAP cells -dox (yellow) and in wtSRCAP cells +dox (blue) at time-lapse conditions shown on top and survival time functions obtained with GRID (black lines). cdSRCAP+dox: 1623 tracks in 16 cells, cdSRCAP-dox: 4624 tracks in 47 cells, wtSRCAP+dox: 2571 tracks in 34 cells. **n,** State spectra of dissociation rates obtained with GRID from distributions in (m) using all data for Halo-NANOG in cdSRCAP cells +dox (red, left panel), in cdSRCAP cells -dox (yellow, middle panel), and in wtSRCAP cells +dox (blue, right panel). As an error estimation GRID was run 500 times with each run using 80 % of the data (black circles). The dashed lines indicate a dissociation rate of 0.02 $s^{-1}$. **o,** Average residence time of Halo-NANOG molecules bound longer than 50s, calculated from data displayed in fig.S7-l,n, with the statistics provided there. Error bars denote s.d. of 500 resamplings with randomly selected 80 % of the data and error propagation. **p,** Enrichment of OCT4 and SOX2 (left panel) and accessibility score determined by ATAC-Seq in SYAT cells treated with CTRL or IAA for 4 hours at NANOG peaks shared between CTRL and IAA condition or unique to 4 hour-IAA treatment.

**Table S1. List of primers used in the study**

| Primer Name | Source or reference | Sequence | Application |
|---|---|---|---|
| Rps5 Fw | This paper | TGGCAGAGACCCCTGACAT | RTqPCR |
| Rps5 Rev | This paper | GGGCAGGTACTTGGCATACT | RTqPCR |
| Zfp42 Fw | This paper | CCCTCGACAGACTGACCCTAA | RTqPCR |
| Zfp42 Rev | This paper | TCGGGGCTAATCTCACTTTCAT | RTqPCR |
| Pou5f1 Fw | This paper | GAGGAGTCCCAGGACATGAA | RTqPCR |
| Pou5f1 Rev | This paper | AGATGGTGGTCTGGCTGAAC | RTqPCR |
| Sox2 Fw | This paper | GCACATGAACGGCTGGAGCAACG | RTqPCR |
| Sox2 Rev | This paper | TGCTGCGAGTAGGACATGCTGTAGG | RTqPCR |
| Nanog Fw | This paper | AACCAAAGGATGAAGTGCAAGCGG | RTqPCR |
| Nanog Rev | This paper | TCCAAGTTGGGTTGGTCCAAGTCT | RTqPCR |
| Esrrb Fw | This paper | CCGATTTCCCCACCTGCTAA | RTqPCR |
| Esrrb Rev | This paper | GGCCCAGTTGATGAGGAACA | RTqPCR |
| c-Myc Fw | This paper | GAGCTCCTCGAGCTGTTTGA | RTqPCR |
| c-Myc rev | This paper | GCATCGTCGTGGCTGTCT | RTqPCR |
| SRCAP gRNA Fw | This paper | CACCGCGAAAGGCCAAGACGTGAat | gRNA |
| SRCAP gRNA rev | This paper | AAACatTCACGTCTTGGCCTTTCGC | gRNA |
| SRCAP homology arms Fw | This paper | cccacctatgtatccctactattagtcacagaggtcgaa aggccaagacgGGTGGCGGTGGATCCGG AG | PCR of KI cassette |
| SRCAP homology arms Rev | This paper | gcaagggaaagcggccatggtgggatacataagtga aggggctgcccatTCAttattcctttgccctcggacg | PCR of KI cassette |
| gDNA SRCAP Fw | This paper | GTAGAGGAGTCAGAGGTAGAAG | Validation of KI on gDNA |
| gDNA SRCAP Rev | This paper | TGGTGGAAAGGAAGACAGC | Validation of KI on gDNA |
| Ypet Rev | This paper | GGAGACGTGGAGGAGAAC | Validation of KI on gDNA |
| P2A Fw | This paper | CGTTGTGGCTGTTGTAGTTG | Validation of KI on gDNA |
| SRCAP Fragment 1 Fw | This paper | CTACCCTCGTAAAACCGGTCGACatgca gagcagcccctccac | Cloning of wt and cd SRCAP |
| wtSRCAP Fragment 1 Rev | This paper | gccttcttgcttgggcactggctgcagcattctgttgacctt c | Cloning of wtSRCAP |
| cdSRCAP Fragment 1 Rev | This paper | ctgagcCGCAGCcagaataag | Cloning of cdSRCAP |
| wtSRCAP Fragment 2 Fw | This paper | gccagtgcccaagcaagaaggc | Cloning of wtSRCAP |
| wtSRCAP Fragment 2 Rev | This paper | gaagcagaatatgcgtttgtccgc | Cloning of wtSRCAP |
| cdSRCAP Fragment 2 Fw | This paper | ctgGCTGCGgctcagaacatcGCGaatttcaagt cccaacg | Cloning of cdSRCAP |
| cdSRCAP Fragment 2 Rev | This paper | ccactggagaacttggaggcag | Cloning of cdSRCAP |
| wtSRCAP Fragment 3 Fw | This paper | gcggacaaacgcatattctgcttcatcctttcaactcgaa gtg | Cloning of wtSRCAP |
| cdSRCAP Fragment 3 Fw | This paper | gcctccaagttctccagtgggcccagctccaggtcaca cactg | Cloning of cdSRCAP |
| SRCAP Fragment 3 Rev | This paper | CGCCCTTGCTCACCATGTCGACTCCcgt cttggcctttcgacctctgtg | Cloning of wt and cd SRCAP |
| TRE3G Fw | This paper | gtcttataccaactttccgt | Screening of SRCAP vectors |
| SRCAP Rev | This paper | cacattgctccagaactgcc | Screening of SRCAP vectors |
| SRCAP Fw | This paper | cctgacaccactcctagcctg | Screening of SRCAP vectors |
| mCherry Rev | This paper | gccgttcacggagccctcc | Screening of SRCAP vectors |

**Table S2. List of pioneer transcription factors**

| Name | Reference |
|---|---|
| AP-1 | Lemma et al. Epigenetics and Chromatin 2022 |
| ASCL1 | Lemma et al. Epigenetics and Chromatin 2022 |
| ATF4 | Peng et al. eLife 2024 |
| BACH1 | Peng et al. eLife 2024 |
| BCL6 | Peng et al. eLife 2024 |
| CEBPA | Peng et al. eLife 2024 |
| CEBPB | Peng et al. eLife 2024 |
| CEBPD | Peng et al. eLife 2024 |
| CEBPG | Peng et al. eLife 2024 |
| FLI1 | Lemma et al. Epigenetics and Chromatin 2022 |
| FOS | Peng et al. eLife 2024 |
| FOSL2 | Peng et al. eLife 2024 |
| FOXA1 | Peng et al. eLife 2024 |
| FOXA2 | Peng et al. eLife 2024 |
| FOXA3 | Peng et al. eLife 2024 |
| FOXD3 | Lemma et al. Epigenetics and Chromatin 2022 |
| FOXE1 | Lemma et al. Epigenetics and Chromatin 2022 |
| FOXK2 | Peng et al. eLife 2024 |
| FOXM1 | Lemma et al. Epigenetics and Chromatin 2022 |
| FOXO1 | Lemma et al. Epigenetics and Chromatin 2022 |
| GATA1 | Peng et al. eLife 2024 |
| GATA2 | Peng et al. eLife 2024 |
| GATA3 | Lemma et al. Epigenetics and Chromatin 2022 |
| GATA4 | Peng et al. eLife 2024 |
| GATA5 | Lemma et al. Epigenetics and Chromatin 2022 |
| GATA6 | Lemma et al. Epigenetics and Chromatin 2022 |
| GRHL1 | Lemma et al. Epigenetics and Chromatin 2022 |
| GRHL2 | Lemma et al. Epigenetics and Chromatin 2022 |
| HLF | Peng et al. eLife 2024 |
| HMBOX1 | Peng et al. eLife 2024 |
| HNF4A | Peng et al. eLife 2024 |
| IKZF1 | Peng et al. eLife 2024 |
| JUNB | Peng et al. eLife 2024 |
| JUND | Peng et al. eLife 2024 |
| KLF4 | Lemma et al. Epigenetics and Chromatin 2022 |
| LEF1 | Peng et al. eLife 2024 |
| MAFF | Peng et al. eLife 2024 |
| MAFG | Peng et al. eLife 2024 |
| MEF2A | Peng et al. eLife 2024 |
| MYB | Lemma et al. Epigenetics and Chromatin 2022 |

| | |
|---|---|
| NFATC3 | Peng et al. eLife 2024 |
| NFE2 | Peng et al. eLife 2024 |
| NFE2L1 | Peng et al. eLife 2024 |
| NFIL3 | Peng et al. eLife 2024 |
| NR2F1 | Peng et al. eLife 2024 |
| NR5A1 | Peng et al. eLife 2024 |
| NF-Ya | Lemma et al. Epigenetics and Chromatin 2022 |
| OTX2 | Lemma et al. Epigenetics and Chromatin 2022 |
| PAX7 | Lemma et al. Epigenetics and Chromatin 2022 |
| PBX1 | Lemma et al. Epigenetics and Chromatin 2022 |
| PBX2 | Peng et al. eLife 2024 |
| POU5F1 | Lemma et al. Epigenetics and Chromatin 2022 |
| PPARG | Peng et al. eLife 2024 |
| RUNX1 | Lemma et al. Epigenetics and Chromatin 2022 |
| RUNX3 | Lemma et al. Epigenetics and Chromatin 2022 |
| RXRB | Peng et al. eLife 2024 |
| SOX2 | Lemma et al. Epigenetics and Chromatin 2022 |
| SOX9 | Lemma et al. Epigenetics and Chromatin 2022 |
| SPI1 | Lemma et al. Epigenetics and Chromatin 2022 |
| SRF | Peng et al. eLife 2024 |
| STAT3 | Peng et al. eLife 2024 |
| TEAD1 | Peng et al. eLife 2024 |
| TFAP2C | Lemma et al. Epigenetics and Chromatin 2022 |
| TP53 | Lemma et al. Epigenetics and Chromatin 2022 |
| TP63 | Lemma et al. Epigenetics and Chromatin 2022 |
| ZNF24 | Peng et al. eLife 2024 |
