# Supplementary material for: Mechanisms of gene regulation by SRCAP and H2A.Z
Source: Nat Commun. 2026 Mar 6;17:3560. doi: 10.1038/s41467-026-70087-x (PMC13087030; doi:10.1038/s41467-026-70087-x)
Supplement: Supplementary file 2 — Description of Additional Supplementary Files [file 41467_2026_70087_MOESM2_ESM.pdf]

Description of Additional Supplementary Files

Supplementary Data 1: Excel file containing RNA-Seq raw counts and differential analysis results

Supplementary Data 2: Excel file containing results from proteome and chromatome analysis

Supplementary Data 3: Excel file containing results from TF footprinting analysis

Supplementary Data 4: Number of nucleosomes quantified in MNase analysis presented in Fig.4 and S4

Supplementary Data 5: Results of statistical tests performed in the study
