# Supplementary material for: Mechanisms of gene regulation by SRCAP and H2A.Z
Source: Nat Commun. 2026 Mar 6;17:3560. doi: 10.1038/s41467-026-70087-x (PMC13087030; doi:10.1038/s41467-026-70087-x)
Supplement: Supplementary file 8 — Reporting Summary [file 41467_2026_70087_MOESM8_ESM.pdf]

nature portfolio

Corresponding author(s): David Suter

Last updated by author(s): Jan 9, 2026

Reporting Summary

Nature Portfolio wishes to improve the reproducibility of the work that we publish. This form provides structure for consistency and transparency in reporting. For further information on Nature Portfolio policies, see our Editorial Policies and the Editorial Policy Checklist.

Statistics

For all statistical analyses, confirm that the following items are present in the figure legend, table legend, main text, or Methods section.

n/a	Confirmed	
☐	☒	The exact sample size (*n*) for each experimental group/condition, given as a discrete number and unit of measurement
☐	☒	A statement on whether measurements were taken from distinct samples or whether the same sample was measured repeatedly
☐	☒	The statistical test(s) used AND whether they are one- or two-sided<br>*Only common tests should be described solely by name; describe more complex techniques in the Methods section.*
☐	☒	A description of all covariates tested
☒	☐	A description of any assumptions or corrections, such as tests of normality and adjustment for multiple comparisons
☐	☒	A full description of the statistical parameters including central tendency (e.g. means) or other basic estimates (e.g. regression coefficient) AND variation (e.g. standard deviation) or associated estimates of uncertainty (e.g. confidence intervals)
☐	☒	For null hypothesis testing, the test statistic (e.g. *F*, *t*, *r*) with confidence intervals, effect sizes, degrees of freedom and *P* value noted<br>*Give P values as exact values whenever suitable.*
☒	☐	For Bayesian analysis, information on the choice of priors and Markov chain Monte Carlo settings
☒	☐	For hierarchical and complex designs, identification of the appropriate level for tests and full reporting of outcomes
☐	☒	Estimates of effect sizes (e.g. Cohen's *d*, Pearson's *r*), indicating how they were calculated

Our web collection on statistics for biologists contains articles on many of the points above.

Software and code

Policy information about availability of computer code

Data collection	Images of SRCAP depletion and H2A.Z mitotic association were acquired using IN Cell Analyzer 2200 v7.1. Flow Cytometry data were acquired using BD FACSDiva software v6.1.3. qPCR data were acquired using QuantStudio Software v1.3
Data analysis	For data analysis, we used R studio Version 2023.12.0+369 with R packages Cluster Profiler Version 4.10.1, ggplot2 Version 3.5.0, ggrepel Version 0.9.5, biomaRt Version 2.58.2, DeSeq2 Version 1.42.1, edgeR Version 4.0.11, limma Version 3.58.1, MethylKit Version 1.28.0. For the primary analysis of genomic data, we used STAR Version 2.7.6a, Picard Version 2.23.7, MACS Version 3.0.0a7, DeepTools Version 3.5.1, Bedops Version 2.4.41, BEDtools Version 2.30.0, SAMtools Version 1.14, DANPOS2 Version 3.1.1, Bwa-Meth, MethylDackel Version 0.5.3, TOBIAS Version 0.13.3, SEA Version 5.5.5. For calculation of solvant accessible surface from SRCAP structure, FreeSASA Version 2.1.2 was used. For image analysis, we used FiJi Version 2.14.0/1.54f.

For manuscripts utilizing custom algorithms or software that are central to the research but not yet described in published literature, software must be made available to editors and reviewers. We strongly encourage code deposition in a community repository (e.g. GitHub). See the Nature Portfolio guidelines for submitting code & software for further information.

Data

Policy information about availability of data

All manuscripts must include a data availability statement. This statement should provide the following information, where applicable:

- Accession codes, unique identifiers, or web links for publicly available datasets
- A description of any restrictions on data availability
- For clinical datasets or third party data, please ensure that the statement adheres to our policy

All sequencing data were deposited at NCBI Gene Expression Omnibus (GEO), accession number GSE269310 (with token grkzuqcwplmnlkr).
Proteomics data were deposited at ProteomeXchange, accession number PXD052934 (with token bwU5076lLymG).
Source Data are provided with this paper.

Research involving human participants, their data, or biological material

Policy information about studies with human participants or human data. See also policy information about sex, gender (identity/presentation), and sexual orientation and race, ethnicity and racism.

Reporting on sex and gender	*Use the terms sex (biological attribute) and gender (shaped by social and cultural circumstances) carefully in order to avoid confusing both terms. Indicate if findings apply to only one sex or gender; describe whether sex and gender were considered in study design; whether sex and/or gender was determined based on self-reporting or assigned and methods used. Provide in the source data disaggregated sex and gender data, where this information has been collected, and if consent has been obtained for sharing of individual-level data; provide overall numbers in this Reporting Summary. Please state if this information has not been collected. Report sex- and gender-based analyses where performed, justify reasons for lack of sex- and gender-based analysis.*
Reporting on race, ethnicity, or other socially relevant groupings	*Please specify the socially constructed or socially relevant categorization variable(s) used in your manuscript and explain why they were used. Please note that such variables should not be used as proxies for other socially constructed/relevant variables (for example, race or ethnicity should not be used as a proxy for socioeconomic status). Provide clear definitions of the relevant terms used, how they were provided (by the participants/respondents, the researchers, or third parties), and the method(s) used to classify people into the different categories (e.g. self-report, census or administrative data, social media data, etc.) Please provide details about how you controlled for confounding variables in your analyses.*
Population characteristics	*Describe the covariate-relevant population characteristics of the human research participants (e.g. age, genotypic information, past and current diagnosis and treatment categories). If you filled out the behavioural & social sciences study design questions and have nothing to add here, write "See above."*
Recruitment	*Describe how participants were recruited. Outline any potential self-selection bias or other biases that may be present and how these are likely to impact results.*
Ethics oversight	*Identify the organization(s) that approved the study protocol.*

Note that full information on the approval of the study protocol must also be provided in the manuscript.

Field-specific reporting

Please select the one below that is the best fit for your research. If you are not sure, read the appropriate sections before making your selection.

☒ Life sciences ☐ Behavioural & social sciences ☐ Ecological, evolutionary & environmental sciences

For a reference copy of the document with all sections, see nature.com/documents/nr-reporting-summary-flat.pdf

Life sciences study design

All studies must disclose on these points even when the disclosure is negative.

Sample size	No sample size calculation was performed. Sample size is determined by the genomic regions enriched for H2A.Z. Given the amount of genomic regions studied in each experiment and the parallele approaches that were used in our study (ChIP-Seq, Mass spectrometry, Footprinting prediction etc...), we are confident that our claims are supported by the data.
Data exclusions	No data were excluded from the study.
Replication	We performed experiments on biological duplicates or triplicates from cells cultured and treated independently. For genomic experiments (ChIP-Seq, ATAC-Seq, etc...), we verified the correlation of enrichment scores in enriched regions between replicates before calculating averaged score. Replicates for all experiments were well correlated.
Randomization	Randomization was not relevant to this study, as the experiments were not conducted on defined populations or treatment groups.

Blinding

This does not apply to our study as it is not conducted on groups of patients or animal and no bias can be introduced by the person doing the experiment or analysing it.

Reporting for specific materials, systems and methods

We require information from authors about some types of materials, experimental systems and methods used in many studies. Here, indicate whether each material, system or method listed is relevant to your study. If you are not sure if a list item applies to your research, read the appropriate section before selecting a response.

Materials & experimental systems

n/a	Involved in the study
☐	☒ Antibodies
☐	☒ Eukaryotic cell lines
☐	☐ Palaeontology and archaeology
☐	☐ Animals and other organisms
☐	☐ Clinical data
☐	☐ Dual use research of concern
☐	☐ Plants

Methods

n/a	Involved in the study
☐	☒ ChIP-seq
☒	☐ Flow cytometry
☒	☐ MRI-based neuroimaging

Antibodies

Antibodies used

Rabbit anti-H2A.Z (Abcam #ab4174)(dilution IF 1:1000, ChIP-Seq 1:100), Acetyl-histone H2A.Z (K4,K7,K11) (ThermoFisher Scientific #PA540095 (dilution ChIP-Seq 1:200), Rabbit anti-H2A (Abcam #ab18255) (dilution ChIP-Seq 1:100), Rabbit anti-GFP (Abcam #ab290) (Dilution CUT&Tag 1:50), Rabbit anti histone H3K4me3 (Abcam #ab8580)(Dilution ChIP-Seq 1:100), Rabbit anti histone H3K27me3 (Abcam #ab195477)(Diltution ChIP-Seq 1:200), Rabbit anti histone H2AK119ub1 (Cell Signaling Technology #8240)(Dilution ChIP-Seq 1:200), Rabbit anti-YY1 (Cell Signaling Technology #46395)(Dilution ChIP-Seq 1:50), Mouse anti NF-Ya (Santa-Cruz Biotechnology #sc-17753)(Dilution ChIP-Seq 1:150), Rabbit anti-Oct4 (Cell Signaling Technology #5677)(Dilution ChIP-Seq 1:60), Rabbit anti-Sox2 (Cell Signaling Technology #23064)(Dilution ChIP-Seq 1:60), Rabbit anti-Nanog (Cell Signaling Technology #8822)(Dilution ChIP-Seq 1:100), Rabbit anti-SRCAP (Kerafast #ESL103)(Dilution WB 1:1000, CUT&RUN 1:50).

Validation

https://www.abcam.com/en-us/products/primary-antibodies/histone-h2az-antibody-chip-grade-ab4174
https://www.thermofisher.com/antibody/product/H2A-Zac-pan-acetyl-K4-K7-K11-Antibody-Polyclonal/PA5-40095
https://www.abcam.com/en-us/products/primary-antibodies/histone-h2a-antibody-chip-grade-ab18255
https://www.abcam.com/en-us/products/primary-antibodies/gfp-antibody-ab290
https://www.abcam.com/en-us/products/primary-antibodies/histone-h3-tri-methyl-k4-antibody-chip-grade-ab8580
https://www.abcam.com/en-us/products/primary-antibodies/histone-h3-tri-methyl-k27-antibody-chip-grade-ab195477
https://www.cellsignal.com/products/primary-antibodies/ubiquityl-histone-h2a-lys119-d27c4-rabbit-monoclonal-antibody/8240
https://www.cellsignal.com/products/primary-antibodies/yy1-d5d9z-rabbit-monoclonal-antibody/46395
https://www.scbt.com/p/nf-ya-antibody-g-2
https://www.cellsignal.com/products/primary-antibodies/oct-4a-c30a3c1-rabbit-monoclonal-antibody-chip-formulated/5677
https://www.cellsignal.com/products/primary-antibodies/sox2-d9b8n-rabbit-monoclonal-antibody/23064
https://www.cellsignal.com/products/primary-antibodies/nanog-d2a3-rabbit-monoclonal-antibody/8822
https://www.kerafast.com/item/744/anti-snf2-related-cbp-activator-protein-srcap-antibody-affinity-purified

Eukaryotic cell lines

Policy information about cell lines and Sex and Gender in Research

Cell line source(s)

CGR8 mESCs were purchased from Sigma Aldrich (#I5148)
SYAT and SYA cell lines were derived from CGR8 for this study
HEK-293T cells: ATCC

Authentication

CGR8 and HEK-293T were authenticated by supplier

Mycoplasma contamination

All cell lines were tested for absence of Mycoplasma contamination every 6 months and prior establishment of new cell lines.

Commonly misidentified lines
(See ICLAC register)

HEK-293T: these cells were used for lentiviral vector production

Palaeontology and Archaeology

Specimen provenance

Provide provenance information for specimens and describe permits that were obtained for the work (including the name of the issuing authority, the date of issue, and any identifying information). Permits should encompass collection and, where applicable, export.

Specimen deposition	*Indicate where the specimens have been deposited to permit free access by other researchers.*
Dating methods	*If new dates are provided, describe how they were obtained (e.g. collection, storage, sample pretreatment and measurement), where they were obtained (i.e. lab name), the calibration program and the protocol for quality assurance OR state that no new dates are provided.*

☐ Tick this box to confirm that the raw and calibrated dates are available in the paper or in Supplementary Information.

Ethics oversight	*Identify the organization(s) that approved or provided guidance on the study protocol, OR state that no ethical approval or guidance was required and explain why not.*

Note that full information on the approval of the study protocol must also be provided in the manuscript.

Animals and other research organisms

Policy information about studies involving animals; ARRIVE guidelines recommended for reporting animal research, and Sex and Gender in Research

Laboratory animals	*For laboratory animals, report species, strain and age OR state that the study did not involve laboratory animals.*
Wild animals	*Provide details on animals observed in or captured in the field; report species and age where possible. Describe how animals were caught and transported and what happened to captive animals after the study (if killed, explain why and describe method; if released, say where and when) OR state that the study did not involve wild animals.*
Reporting on sex	*Indicate if findings apply to only one sex; describe whether sex was considered in study design, methods used for assigning sex. Provide data disaggregated for sex where this information has been collected in the source data as appropriate; provide overall numbers in this Reporting Summary. Please state if this information has not been collected. Report sex-based analyses where performed, justify reasons for lack of sex-based analysis.*
Field-collected samples	*For laboratory work with field-collected samples, describe all relevant parameters such as housing, maintenance, temperature, photoperiod and end-of-experiment protocol OR state that the study did not involve samples collected from the field.*
Ethics oversight	*Identify the organization(s) that approved or provided guidance on the study protocol, OR state that no ethical approval or guidance was required and explain why not.*

Note that full information on the approval of the study protocol must also be provided in the manuscript.

Clinical data

Policy information about clinical studies

All manuscripts should comply with the ICMJE guidelines for publication of clinical research and a completed CONSORT checklist must be included with all submissions.

Clinical trial registration	*Provide the trial registration number from ClinicalTrials.gov or an equivalent agency.*
Study protocol	*Note where the full trial protocol can be accessed OR if not available, explain why.*
Data collection	*Describe the settings and locales of data collection, noting the time periods of recruitment and data collection.*
Outcomes	*Describe how you pre-defined primary and secondary outcome measures and how you assessed these measures.*

Dual use research of concern

Policy information about dual use research of concern

Hazards

Could the accidental, deliberate or reckless misuse of agents or technologies generated in the work, or the application of information presented in the manuscript, pose a threat to:

No	Yes	
☒	☐	Public health
☒	☐	National security
☒	☐	Crops and/or livestock
☒	☐	Ecosystems
☒	☐	Any other significant area

Experiments of concern

Does the work involve any of these experiments of concern:

No	Yes	
☒	☐	Demonstrate how to render a vaccine ineffective
☒	☐	Confer resistance to therapeutically useful antibiotics or antiviral agents
☒	☐	Enhance the virulence of a pathogen or render a nonpathogen virulent
☒	☐	Increase transmissibility of a pathogen
☒	☐	Alter the host range of a pathogen
☒	☐	Enable evasion of diagnostic/detection modalities
☒	☐	Enable the weaponization of a biological agent or toxin
☒	☐	Any other potentially harmful combination of experiments and agents

Plants

Seed stocks	*Report on the source of all seed stocks or other plant material used. If applicable, state the seed stock centre and catalogue number. If plant specimens were collected from the field, describe the collection location, date and sampling procedures.*
Novel plant genotypes	*Describe the methods by which all novel plant genotypes were produced. This includes those generated by transgenic approaches, gene editing, chemical/radiation-based mutagenesis and hybridization. For transgenic lines, describe the transformation method, the number of independent lines analyzed and the generation upon which experiments were performed. For gene-edited lines, describe the editor used, the endogenous sequence targeted for editing, the targeting guide RNA sequence (if applicable) and how the editor was applied.*
Authentication	*Describe any authentication procedures for each seed stock used or novel genotype generated. Describe any experiments used to assess the effect of a mutation and, where applicable, how potential secondary effects (e.g. second site T-DNA insertions, mosiacism, off-target gene editing) were examined.*

ChIP-seq

Data deposition

☒ Confirm that both raw and final processed data have been deposited in a public database such as GEO.

☒ Confirm that you have deposited or provided access to graph files (e.g. BED files) for the called peaks.

Data access links
May remain private before publication.

GSE269310 : https://www.ncbi.nlm.nih.gov/geo/query/acc.cgi?acc=GSE269310
reviewer token grkzuqcwplmnlkr

Files in database submission

Raw fastq files for each replicate available in GEO. Additionally, processed files listed below:
SYAT_H2AZ_unt_m.bw
SYAT_H2AZ_IAA2h_1_m.bw
SYAT_H2AZ_IAA2h_2_m.bw
SYAT_H2AZ_IAA4h_1_m.bw
SYAT_H2AZ_IAA4h_2_m.bw
SYAT_H2AZ_IAA6h_1_m.bw
SYAT_H2AZ_IAA6h_2_m.bw
SYAT_H2AZ_IAA8h_1_m.bw
SYAT_H2AZ_IAA8h_2_m.bw
SYAT_unt_H2A_1.bw
SYAT_IAA2h_H2A_1.bw
SYAT_IAA4h_H2A_1.bw
SYAT_IAA6h_H2A_1.bw
SYAT_IAA8h_H2A_1.bw
SYAT_unt_H2A_2.bw
SYAT_IAA2h_H2A_2.bw
SYAT_IAA4h_H2A_2.bw
SYAT_IAA6h_H2A_2.bw
SYAT_IAA8h_H2A_2.bw
AS_H2AZ_m_1.bw
M_H2AZ_m_1.bw
EG1_H2AZ_m_1.bw
LG1_H2AZ_m_1.bw
S_H2AZ_m_1.bw
G2_H2AZ_m_1.bw
AS_H2AZ_m_2.bw
M_H2AZ_m_2.bw
EG1_H2AZ_m_2.bw

LG1_H2AZ_m_2.bw
S_H2AZ_m_2.bw
G2_H2AZ_m_2.bw
acH2AZ_AS_1.bw
acH2AZ_AS_2.bw
acH2AZ_M_1.bw
acH2AZ_M_2.bw
AS_unt_H2AZ_m.bw
AS_IAA_H2AZ_m.bw
M_unt_H2AZ_m.bw
M_IAA_H2AZ_m.bw
ZHBTc4_unt_H2AZ_1.bw
ZHBTc4_Dox9h_H2AZ_1.bw
ZHBTc4_Dox12h_H2AZ_1.bw
ZHBTc4_unt_H2AZ_2.bw
ZHBTc4_Dox9h_H2AZ_2.bw
ZHBTc4_Dox12h_H2AZ_2.bw
SYAT_unt_YY1_1.bw
SYAT_IAA4h_YY1_1.bw
SYAT_IAA6h_YY1_1.bw
SYAT_IAA8h_YY1_1.bw
SYAT_unt_Oct4_1.bw
SYAT_IAA4h_Oct4_1.bw
SYAT_IAA6h_Oct4_1.bw
SYAT_IAA8h_Oct4_1.bw
SYAT_unt_Oct4_2.bw
SYAT_IAA4h_Oct4_2.bw
SYAT_IAA6h_Oct4_2.bw
SYAT_IAA8h_Oct4_2.bw
SYAT_unt_Sox2_1.bw
SYAT_IAA4h_Sox2_1.bw
SYAT_IAA6h_Sox2_1.bw
SYAT_IAA8h_Sox2_1.bw
SYAT_unt_Sox2_2.bw
SYAT_IAA4h_Sox2_2.bw
SYAT_IAA6h_Sox2_2.bw
SYAT_IAA8h_Sox2_2.bw
SYAT_Unt_Nanog_1.bw
SYAT_IAA4h_Nanog_1.bw
SYAT_IAA6h_Nanog_1.bw
SYAT_IAA8h_Nanog_1.bw
SYAT_unt_NFYa_1.bw
SYAT_IAA4h_NFYa_1.bw
SYAT_IAA6h_NFYa_1.bw
SYAT_IAA8h_NFYa_1.bw
SYAT_H2AZ_unt_m_peaks.bed
SYAT_H2AZ_IAA2h_1_m_peaks.bed
SYAT_H2AZ_IAA2h_2_m_peaks.bed
SYAT_H2AZ_IAA4h_1_m_peaks.bed
SYAT_H2AZ_IAA4h_2_m_peaks.bed
SYAT_H2AZ_IAA6h_1_m_peaks.bed
SYAT_H2AZ_IAA6h_2_m_peaks.bed
SYAT_H2AZ_IAA8h_1_m_peaks.bed
SYAT_H2AZ_IAA8h_2_m_peaks.bed
AS_H2AZ_m_1_peaks.bed
M_H2AZ_m_1_peaks.bed
EG1_H2AZ_m_1_peaks.bed
LG1_H2AZ_m_1_peaks.bed
S_H2AZ_m_1_peaks.bed
G2_H2AZ_m_1_peaks.bed
AS_H2AZ_m_2_peaks.bed
M_H2AZ_m_2_peaks.bed
EG1_H2AZ_m_2_peaks.bed
LG1_H2AZ_m_2_peaks.bed
S_H2AZ_m_2_peaks.bed
G2_H2AZ_m_2_peaks.bed
acH2AZ_AS_1_peaks.bed
acH2AZ_AS_2_peaks.bed
acH2AZ_M_1_peaks.bed
acH2AZ_M_2_peaks.bed
AS_unt_H2AZ_m_peaks.bed
AS_IAA_H2AZ_m_peaks.bed
M_unt_H2AZ_m_peaks.bed
M_IAA_H2AZ_m_peaks.bed
ZHBTc4_unt_H2AZ_1_peaks.bed
ZHBTc4_Dox9h_H2AZ_1_peaks.bed

ZHBTc4_Dox12h_H2AZ_1_peaks.bed
ZHBTc4_unt_H2AZ_2_peaks.bed
ZHBTc4_Dox9h_H2AZ_2_peaks.bed
ZHBTc4_Dox12h_H2AZ_2_peaks.bed
SYAT_unt_YY1_1_peaks.bed
SYAT_IAA4h_YY1_1_peaks.bed
SYAT_IAA6h_YY1_1_peaks.bed
SYAT_IAA8h_YY1_1_peaks.bed
SYAT_unt_Oct4_1_peaks.bed
SYAT_IAA4h_Oct4_1_peaks.bed
SYAT_IAA6h_Oct4_1_peaks.bed
SYAT_IAA8h_Oct4_1_peaks.bed
SYAT_unt_Oct4_2_peaks.bed
SYAT_IAA4h_Oct4_2_peaks.bed
SYAT_IAA6h_Oct4_2_peaks.bed
SYAT_IAA8h_Oct4_2_peaks.bed
SYAT_unt_Sox2_1_peaks.bed
SYAT_IAA4h_Sox2_1_peaks.bed
SYAT_IAA6h_Sox2_1_peaks.bed
SYAT_IAA8h_Sox2_1_peaks.bed
SYAT_unt_Sox2_2_peaks.bed
SYAT_IAA4h_Sox2_2_peaks.bed
SYAT_IAA6h_Sox2_2_peaks.bed
SYAT_IAA8h_Sox2_2_peaks.bed
SYAT_Unt_Nanog_1_peaks.bed
SYAT_IAA4h_Nanog_1_peaks.bed
SYAT_IAA6h_Nanog_1_peaks.bed
SYAT_IAA8h_Nanog_1_peaks.bed
SYAT_unt_NFYa_1_peaks.bed
SYAT_IAA4h_NFYa_1_peaks.bed
SYAT_IAA6h_NFYa_1_peaks.bed
SYAT_IAA8h_NFYa_1_peaks.bed

Genome browser session
(e.g. UCSC)

No longer applicable

Methodology

Replicates

ChIP-Seq experiments against H2A.Z, H2A, H3K4me3, OCT4 and SOX2 were performed in biological duplicates, except for the ChIP-seq against H2A.Z after 1h of IAA treatment in mitotic versus asynchronous cells that were performed as single replicates. ChIP-Seq against H3K27me3, H2AK119ub1, NANOG, YY1 and NF-Ya were performed as single replicates.

Sequencing depth

All sequencing files were obtained from paired-end sequencing with 75 PE reads
Sample name % Uniquely mapped reads Total number of reads
AS_H2AZ_m_1 80.74 37408908
AS_Inp_m_1 76.43 35630363
EG1_H2AZ_m_1 81.89 40799428
EG1_Inp_m_1 61.1 32708860
G2_H2AZ_m_1 81.61 45835836
G2_Inp_m_1 74.08 38944086
LG1_H2AZ_m_1 82.18 39997303
LG1_Inp_m_1 79.65 36761980
M_H2AZ_m_1 80.53 44804601
M_Inp_m_1 77.19 39364309
S_H2AZ_m_1 82.73 46848270
S_Inp_m_1 68.38 29964284
AS_IAA_H2AZ_m_1 77.41 53808085
AS_unt_H2AZ_m_1 77.85 57011666
M_IAA_H2AZ_m_1 77.67 55623101
M_unt_H2AZ_m_1 79.13 55489581
SYAT_IAA4h_Oct4_1 77 56886823
SYAT_IAA4h_Sox2_1 78.92 54340992
SYAT_IAA6h_Oct4_1 76.92 55658107
SYAT_IAA6h_Sox2_1 76.98 61365776
SYAT_IAA8h_Oct4_1 75.37 53134419
SYAT_IAA8h_Sox2_1 74.4 51314040
ZHBTc4_Dox12h_H2AZ_1 78.39 48803345
ZHBTc4_Dox12h_H2AZ_2 73.85 51788087
ZHBTc4_Dox9h_H2AZ_1 77.1 53959369
ZHBTc4_Dox9h_H2AZ_2 73.04 58180797
ZHBTc4_unt_H2AZ_1 77.18 56896743
ZHBTc4_unt_H2AZ_2 77.04 53956280
SYAT_IAA4h_Oct4_2 75.36 45173086
SYAT_IAA4h_Sox2_2 74.66 47686389
SYAT_IAA6h_Oct4_2 76.25 49835436

	SYAT_IAA6h_Sox2_2 72.23 45111827<br>SYAT_IAA8h_Oct4_2 76.59 54223783<br>SYAT_IAA8h_Sox2_2 77.68 52050216<br>SYAT_unt_Oct4_2 76 44541749<br>SYAT_unt_Sox2_2 77.42 42937876<br>SYAT_IAA4h_H3K4me3_1 79.88 54748605<br>SYAT_IAA6h_H3K4me3_1 82.69 50622798<br>SYAT_IAA8h_H3K4me3_1 80.94 52805723<br>SYAT_NT_H3K4me3_1 79.71 57709328<br>SYAT_IAA2h_H2A_2 75.70 47703535<br>SYAT_IAA4h_H2A_2 73.84 55924960<br>SYAT_IAA6h_H2A_2 73.39 53825145<br>SYAT_IAA8h_H2A_2 75.73 60713508<br>SYAT_unt_H2A_2 74.59 42232224<br>SYAT_Unt_Nanog_1 62.89 44183475<br>SYAT_IAA4h_Nanog_1 75.37 42487182<br>SYAT_IAA6h_Nanog_1 76.54 35945053<br>SYAT_IAA8h_Nanog_1 70.07 42495443<br>SYAT_unt_H2Aub_1 83.61 92583726<br>SYAT_IAA4h_H2Aub_1 83.81 80026680<br>SYAT_IAA6h_H2Aub_1 83.53 91027270<br>SYAT_IAA8h_H2Aub_1 83.75 92335613<br>SRCAPwt_high_NT_H2AZ 79.91 55428312<br>SRCAPwt_high_IAA_H2AZ 64.73 59922575<br>SRCAPmut_high_NT_H2AZ 79.25 57048200<br>SRCAPmut_high_IAA_H2AZ 71.54 70821669<br>SRCAPmut_NT_noDox_H2AZ 79.95 46055085<br>SRCAPmut_NT_Dox_H2AZ 78.09 65014753<br>SRCAPmut_IAA_noDox_H2AZ 75.93 55487790<br>SRCAPmut_IAA_Dox_H2AZ 73.21 50931568<br>SYAT_NT_H3K27me3_1 67.84 62756025<br>SYAT_IAA4h_H3K27me3_1 77.17 61549255<br>SYAT_IAA6h_H3K27me3_1 75.43 56291325<br>SYAT_IAA8h_H3K27me3_1 76.75 64836906<br>SYAT_NT_Nanog_2 71.95 59166340<br>SYAT_IAA5uM_Nanog_2 82.88 56437864<br>SYAT_IAA500uM_Nanog_2 82.36 59702521<br>SYAT_IAA500nM_Nanog_2 78.14 64522146<br>SYAT_IAA250nM_Nanog_2 46.84 61625811
Antibodies	Rabbit anti-H2A.Z (Abcam #ab4174)(dilution ChIP-Seq 1:100), Acetyl-histone H2A.Z (K4,K7,K11) (ThermoFisher Scientific #PA540095 (dilution ChIP-Seq 1:200), Rabbit anti-H2A (Abcam #ab18255) (dilution ChIP-Seq 1:100), Rabbit anti-GFP (Abcam #ab290)(Dilution CUT&Tag 1:50), Rabbit anti histone H3K4me3 (Abcam #ab8580)(Dilution ChIP-Seq 1:100), Rabbit anti histone H3K27me3 (Abcam #ab195477)(Diltution ChIP-Seq 1:200), Rabbit anti histone H2AK119ub1 (Cell Signaling Technology #8240)(Dilution ChIP-Seq 1:200), Rabbit anti-YY1 (Cell Signaling Technology #46395)(Dilution ChIP-Seq 1:50), Mouse anti NF-Ya (Santa-Cruz Biotechnology #sc-17753) (Dilution ChIP-Seq 1:150), Rabbit anti-Oct4 (Cell Signaling Technology #5677)(Dilution ChIP-Seq 1:60), Rabbit anti-Sox2 (Cell Signaling Technology #23064)(Dilution ChIP-Seq 1:60), Rabbit anti-Nanog (Cell Signaling Technology #8822)(Dilution ChIP-Seq 1:100), Rabbit anti-SRCAP (Kerafast #ESL103)(CUT&RUN 1:50).
Peak calling parameters	Mapping : STAR --runMode alignReads --alignMatesGapMax 2000 --alignIntronMax 1 --alignEndsType EndToEnd --genomeLoad NoSharedMemory --limitBAMsortRAM 45000000000 --runThreadN 4 --readFilesCommand zcat --outFilterMultimapNmax 1 --genomeDir STARIndexdir --outSAMtype BAM SortedByCoordinate --outFileNamePrefix $outputdir/ --readFilesIn $dir/$sample'_R1_001.fastq' $dir/$sample'_R2_001.fastq'<br>Peak calling : default parameters<br>macs2 callpeak -f BAMPE -t sample.bam -c input.bam -g mm
Data quality	All sequencing yielded 93-95 Q30% values. FastQC was used to check for good sequence quality (no samples were discarded). Duplicate reads were removed. All peaks called are FDR > 5% (q-value 0.05 in MACS2). Our correlations were quality-assured by (i) downsampling reads using normalization factors calculated from Drosophila Spiked In reads, (ii) using a stringent q-value threshold (0.01).
Software	STAR Version 2.7.6a, Picard Version 2.23.7, MACS Version 3.0.0a7, DeepTools Version 3.5.1, Bedops Version 2.4.41, BEDtools Version 2.30.0, SAMtools Version 1.14
